# Manual Khalifa Therapy Improves Functional and Morphological Outcome of Patients with Anterior Cruciate Ligament Rupture in the Knee: A Randomized Controlled Trial

**DOI:** 10.1155/2014/462840

**Published:** 2014-01-30

**Authors:** Michael Ofner, Andreas Kastner, Engelbert Wallenboeck, Robert Pehn, Frank Schneider, Reinhard Groell, Dieter Szolar, Harald Walach, Gerhard Litscher, Andreas Sandner-Kiesling

**Affiliations:** ^1^Department of Sports and Exercise Physiology, University of Vienna, Auf der Schmelz 6, 1150 Vienna, Austria; ^2^Department of Traumatology, General Hospital Linz, 4020 Linz, Austria; ^3^Hospital of Traumatology, 8020 Graz, Austria; ^4^Department of Traumatology, General Hospital Kirchdorf, 4560 Kirchdorf, Austria; ^5^Department of Pediatric Orthopedics, Medical University of Graz, 8036 Graz, Austria; ^6^Institute of Radiology, Medical University of Graz, 8043 Graz, Austria; ^7^Institute of Radiology “Graz South-East”, 8054 Graz, Austria; ^8^Institute of Transcultural Health Studies, European University Viadrina, 15207 Frankfurt Oder, Germany; ^9^Stronach Research Unit for Complementary and Integrative Laser Medicine, Research Unit of Biomedical Engineering in Anesthesia and Intensive Care Medicine, and TCM Research Center Graz, Medical University of Graz, 8036 Graz, Austria; ^10^Department of Anaesthesiology and Intensive Care Medicine, Medical University of Graz, 8036 Graz, Austria

## Abstract

Rupture of the anterior cruciate ligament (ACL) is a high incidence injury usually treated surgically. According to common knowledge, it does not heal spontaneously, although some claim the opposite. Regeneration therapy by Khalifa was developed for injuries of the musculoskeletal system by using specific pressure to the skin. This randomized, controlled, observer-blinded, multicentre study was performed to validate this assumption. Thirty patients with complete ACL rupture, magnetic resonance imaging (MRI) verified, were included. Study examinations (e.g., international knee documentation committee (IKDC) score) were performed at inclusion (*t*_0_). Patients were randomized to receive either standardised physiotherapy (ST) or additionally 1 hour of Khalifa therapy at the first session (STK). Twenty-four hours later, study examinations were performed again (*t*_1_). Three months later control MRI and follow-up examinations were performed (*t*_2_). Initial status was comparable between both groups. There was a highly significant difference of mean IKDC score results at *t*_1_ and *t*_2_. After 3 months, 47% of the STK patients, but no ST patient, demonstrated an end-to-end homogeneous ACL in MRI. Clinical and physical examinations were significantly different in *t*_1_ and *t*_2_. ACL healing can be improved with manual therapy. Physical activity can be performed without pain and nearly normal range of motion after one treatment of specific pressure.

## 1. Introduction

Anterior cruciate ligament (ACL) rupture is an injury of the knee with a high incidence. The optimal management of a torn ACL is still unknown [1]. When associated with knee instability the, injury may limit the level of activity [2, 3]. Conventional knowledge states that the ACL does not heal spontaneously after a complete rupture [4–6]. This leads most surgeons to reconstruct the ACL in symptomatic patients, but neither the correct indication nor the correct time for reconstruction is clear [7, 8]. However, some studies have retrospectively reported spontaneous healing of the ACL after a follow-up assessment of 16–36 months and supported nonoperative treatment [9–12]. Thus, the intriguing question arises, whether the ACL might be able to heal by itself and whether such regeneration can be supported and improved.

Mohamed Khalifa, a therapist from Hallein (Austria) has been working for 30 years with a self-developed manual technique for treating injuries of the musculoskeletal system especially of the knee. For his technique, he applies high pressure to the skin and concomitantly to the structures under the skin (i.e., joints and muscles). International top athletes from various disciplines reported a rapid pain relief and even full recovery, immediately after one-hour treatment of Khalifa therapy (personal communication).

Prior to our study, we evaluated one patient with a complete ACL rupture after a soccer game with magnetic resonance imaging (MRI) and clinical tests (Lachman, Pivot-shift, Anterior-drawer). This patient was physically immobile and reported pain especially when stretching and bending the knee. After one hour of manual therapy, the signs of the injury like the stretching/bending inhibition and pain were gone immediately. Three months after the treatment, an evaluation of the knee with MRI showed an end-to-end continuous ACL with homogeneous signal, and the clinical tests confirmed the stability of the knee.

Encouraged by this finding, the aim of this study was to evaluate if it would be possible to influence the healing of a completely ruptured ACL of the knee as a result of one single special local pressure treatment to the skin for 60 min.

## 2. Material and Methods

This study was designed as a randomized, controlled, observer-blinded, multicentre study running at 4 Austrian hospitals between 2008 and 2011. It was registered at clinicaltrials.gov (ID NCT01762358). The study was approved by the Local Ethics Committee of the Medical University of Graz (EK 19-330 ex 07/08) and meets the requirements of ICH-GCP as well as the requirements of the Declaration of Helsinki.

**Figure 1:**
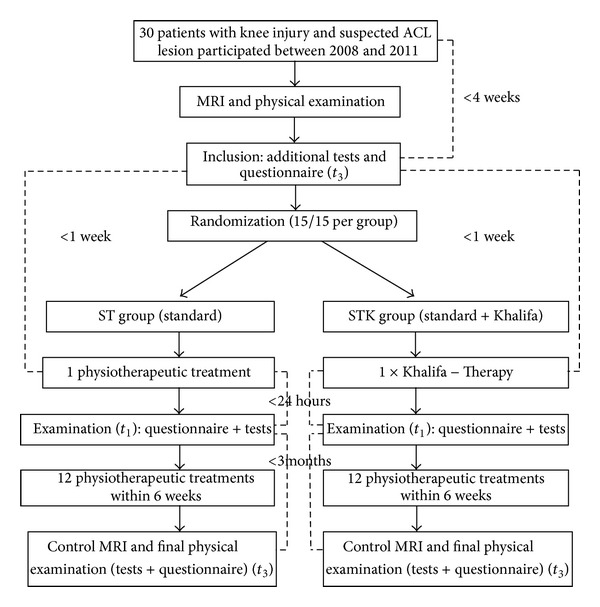
Trial flow. Flow chart of the study including randomization procedure and time intervals.

Patients with suspected ACL lesion were assessed through physical examination (positive Lachman and Pivot-shift test) by a traumatologist or orthopaedic surgeon and sent to MRI to verify an ACL lesion (Figure 1). If both results confirmed the complete ACL rupture and patients met the inclusion and none of the exclusion criteria stated below, they were invited to participate in our study.

### 2.1. Intervention

The therapy introduced in this study is based on different manual therapy techniques (osteopathy, neuromuscular therapy, segment therapy, etc.) and was continuously developed by Mr. Mohamed Khalifa. It is an “impulse-response therapy” aiming to stimulate the self-healing processes. Initially the therapist is looking for an area on the skin with different response to stimuli (in perfusion, colour, etc.) compared to the other tissue. The area/point is called zero point, which is the reference area/point during the therapy. Then he applies pressure in different amplitudes to the skin and concomitantly to the structures under the skin (i.e., joint structures and muscles) around the injured area. This pressure is applied on all segments (dermatomes, myotomes, and osteotomes) that are associated with the injured joint. The force of the pressure is not comparable to that normally used in acupressure in traditional Chinese medicine and it is much higher and at the moment impossible to measure because also frequency plays a significant role (personal communication). The effect of this pressure on the therapy area can be felt on the zero point. This depends a lot on the sensitivity of the therapist which needs to be developed. The injury must be treated as long as it takes to make the zero point as responsive to stimuli as the tissue around it. That takes usually about one hour. It has to be mentioned that side effects (pain during treatment) occur. Currently we are working on programs to teach this therapy to other health care professionals. This study/project aims to objectify the effects of the therapy.

### 2.2. Inclusion Criteria

The study participants had to have a totally ruptured ACL (MRI verified), and the injury should be 4 weeks old as a maximum. Knee function had to be inhibited in at least one respect (stretching, bending, or load). Patients had to be aged between 18 and 45 years, their BMI (body mass index) had to be between 18 and 28, and before the injury, they had to have been athletically active.

### 2.3. Exclusion Criteria

Patients were excluded from the study if they had had any surgical procedures at the injured knee at any previous time. Any acute surgical indication also meant exclusion from the study. The patients should not suffer from diabetes mellitus and/or high blood pressure. Moreover, they should not need any permanent drug treatment.

After receiving written informed consent at inclusion (*t*_0_), each patient completed an 80 items questionnaire including the parameters well-being (0-10), pain (0-10) in numeric rating scales, confidence in their therapists/physicians/radiologists, and the International Knee Documentation Committee (IKDC) Subjective Knee Evaluation Form (score range 0–100) [13, 14].

The following clinical examinations were performed. The knee was inspected visually with regard to axis, signs of inflammation, laterality, and so forth. The maximal flexion/extension of the knee was assessed using the neutral-0-method with goniometer. The Frontal Drawer Test in 90° flexion was performed. In addition, patients underwent the instrumented Lachman Test in 25° flexion with the KT-1000 arthrometer (MEDmetric Corp, San Diego, CA). Side-to-side difference in anterior displacement was recorded in millimetres [15].

The primary variables were the control MRI at *t*_2_ (using the following classification: 1 = End-to-end continuous ACL with homogeneous signal and disappearance of primary and secondary rupture signs; 2 = subtotal ACL rupture, only some continuous fibers or synovial tube showing; 3 = completely ruptured ACL with secondary rupture signs), the KT-1000 test with side-to-side difference in millimetres at *t*_1_ and *t*_2_, and the results of the IKDC score in comparison of *t*_1_ and *t*_2_ to *t*_0_, respectively.

As secondary variables, the changes in range of motion (goniometer), pain, and well-being were assessed as well as the time to return to work.

After the initial assessment was completed, patients were randomized into two treatment groups by using an online tool based on a random algorithm that creates random numbers sequentially (http://www.randomizer.org). The study coordinator informed the patients about the treatment group by phone and told them their individual ID. Additionally, they were instructed not to disclose their treatment group to avoid any unblinding.

Patients received the first treatment within 1 week after inclusion, and they were asked to use crutches until then to avoid further injury. Group ST received 12 units of standardised physiotherapy per study protocol over 6 weeks. Group STK received initially one hour of manual Khalifa therapy (performed by Mohamed Khalifa himself in his private practice in Hallein) followed by 12 units of standardised physiotherapy per study protocol.

Within 24 hours after the first therapy, each patient was re-evaluated with the clinical tests and questionnaire as described above (*t*_1_). Three months later, each patient performed all clinical tests and the questionnaire again plus control MRI in sagittal, axial, and coronal planes, T1, T2 and proton weighted density, with a layer thickness of 2 millimetres to better visualize the ACL (*t*_2_). Two authors (radiologists), blind to patients' group assignment, assessed all MR images independently for primary and secondary signs of ACL rupture on the initial and control MRI. According to Robertson et al., primary signs included ACL morphology and signs of edema, thickening of the ACL, and location of the lesion while secondary signs included bone bruising, posterior cruciate ligament (PCL) buckling, lateral meniscus subluxation, and buckling of the patella tendon [16]. The ACL was considered as completely ruptured when all fibres were ruptured.

### 2.4. Statistical Methods and Analysis

When this study was performed, Khalifa therapy had never been evaluated before. Because of the extraordinary success rates in anecdotal reports after this therapy and assuming a clinically large intergroup effect size, we deemed 15 patients per group sufficient to determine effects of the therapy for this study.

Data preparation was performed using Microsoft Excel 2007. For data analysis, IBM SPSS 16 and Statistica Version 8 were used. Kolmogoroff-Smirnov Test and graphical analyses were used for evaluation of normal distribution. Continuous data was analysed by repeated measure analysis of variance, rank order data was analysed using Wilcoxon signed rank test for changes and Mann-Whitney-*U* test for between group differences. For the frequency between 2 groups, the Fisher exact test was used for paired groups and the chi-square test for unpaired groups with Yates' correction for small cell frequencies. Interobserver reliability of MRI grading was assessed using the Kappa statistic. The level of significance was set at *P* ≤ 0.05. Analysis was performed as intention to treat.

## 3. Results

### 3.1. Demographic

Thirty patients participated in our study, 15 patients per group. Twelve patients were included in centre A, four patients were included in centre B, eight patients were included in centre C and six patients were included in centre D. Results did not differ significantly within the centres. All patients were athletically active before injury. Initial conditions and demographic data showed no differences between both groups (Table 1).

### 3.2. MRI

**Figure 2:**
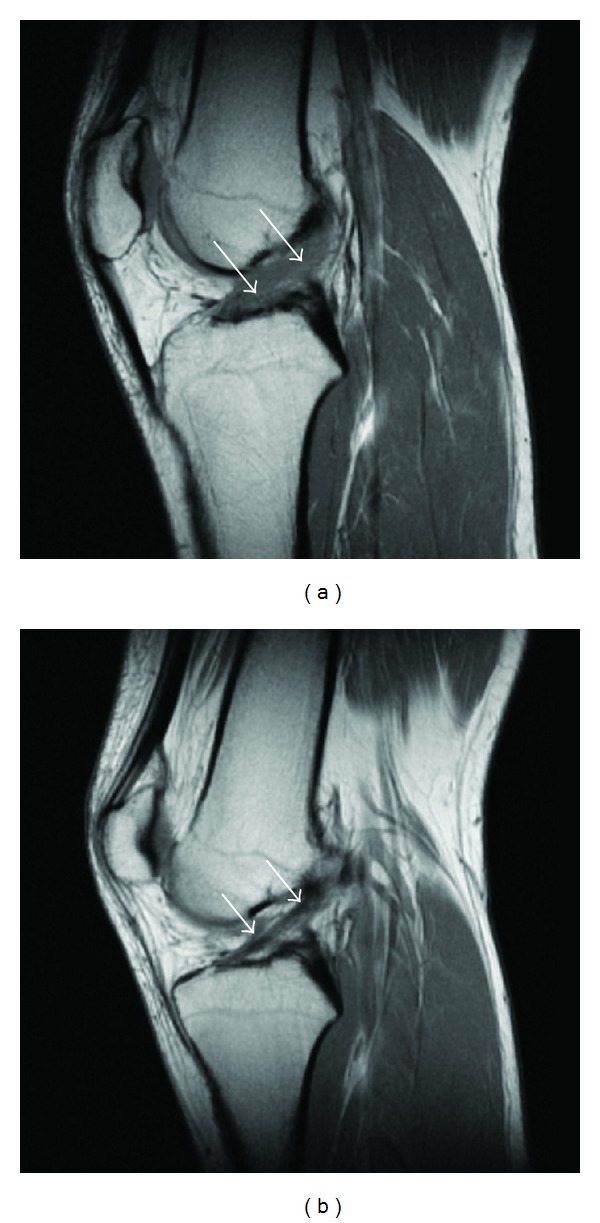
MRI. (a) MRI sagittal view of the knee shows a complete ACL rupture; the arrows point to where the ACL should be. (b) MRI sagittal view of the same patient after Manual Khalifa Therapy and 3 months of follow-up shows a continuous ACL; arrows are pointing out the “new” ACL.

At inclusion, all patients had a complete ACL rupture verified by MRI. Results of control MRI at *t*_2_ differed between both groups. All patients of group ST and 8 patients of group STK still had a complete ACL rupture. The other 7 patients of group STK demonstrated a continuous unsuspected ACL (*P* = 0.01).  Figure 2 shows an MR image of the same patient at inclusion (=*t*_0_, completely ruptured ACL) and 3 months later (=*t*_2_, with an end-to-end ACL). Agreement between both radiologists' MRI grading showed a Kappa result of 0.94.

### 3.3. IKDC and Physical Examination

**Figure 3:**
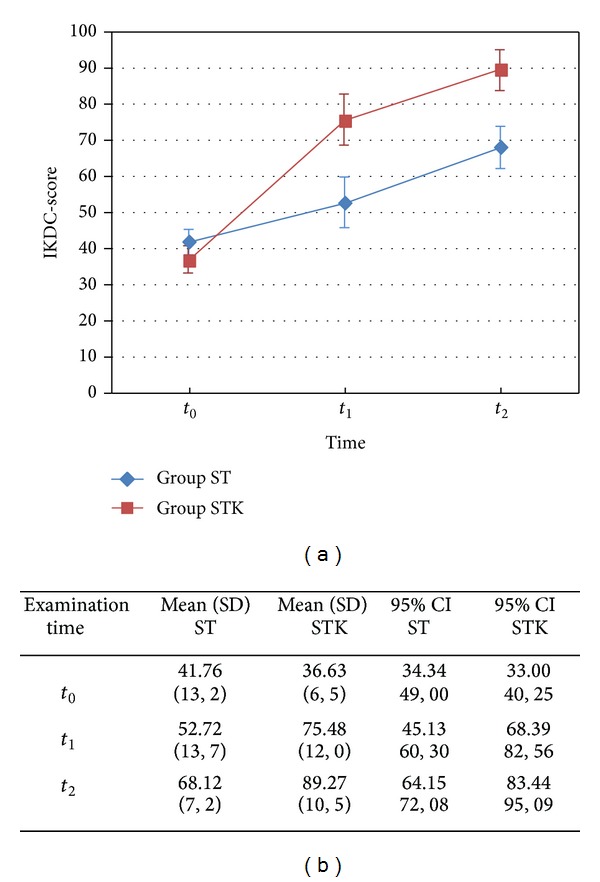
IKDC. (a) Interaction plot of International Knee Documentation Committee (IKDC) Score across three examination dates *t*_0_ (baseline), *t*_1_ (day 1 after 1st treatment), *t*_2_ (3 months after *t*_1_) of standard group (ST), and standard group + Khalifa therapy; vertical bars denote 95% confidence intervals; (b) IKDC mean scores (standard deviations) and 95% confidence Intervals (lower bound; upper bound) per group.

Mean IKDC score (0–100) results showed no significant difference between ST and STK at *t*_0_, but at *t*_1_ and *t*_2_ (*P* < 0.001, Figure 3). Clinical and physical examination, for example, Lachman-KT-1000 test, range of motion, and muscle force, differed between *t*_1_ and *t*_2_ (Table 2).

### 3.4. Secondary Outcomes

Pain and well-being were significantly improved in group STK at *t*_1_ and *t*_2_ (*P* < 0.01, Table 3).

Mean days to return to work after injury were 36 in ST and 13 in STK (*P* < 0.001).

At *t*_2_, 10 patients in group ST but only 2 STK patients considered a surgical repair of the ACL in the near future. Patients' confidence in therapists and physicians did not influence the outcome of the therapy (*P* = 0.46). No side effects of the therapy were reported by the patients.

## 4. Discussion

This is the first randomized controlled study that reports an immediate close-to-normal functional restoration and in 47% of a MRI-verified end-to-end continuous ACL after 3 months after one nonsurgical intervention in patients with an initially verified complete ACL rupture. Manual Khalifa Therapy was developed for treatment of musculoskeletal injuries. Up to now, only two scientific papers exist on this topic. One deals with near infrared spectroscopic data and Khalifa therapy [17] and the other with infrared thermography [18]. However, none of these two papers describes MRI results and clinical outcomes.

ACL rupture is the most relevant injury of the knee. The incidence is approximately 0.5–1 injuries per 1000/year [19]. Arthroscopy of the knee is one of the most frequently applied surgeries worldwide and is often used to treat ACL injuries. Some studies tackle the validity and frequency of these procedures [20]. Additionally, some reports and studies have revealed that the posterior collateral ligament (PCL), medial collateral ligament (MCL), the menisci and the ACL are able to heal spontaneously [5, 9, 10, 13, 21, 22]. If it could be shown that a noninvasive procedure might have equal or even better outcome in the treatment of knee injuries especially of the ACL, it would highly reduce costs and complications at the same time increase patients' quality of life and speed up his functional recovery.

The results of our study confirm the findings of other authors that spontaneous regeneration of ACL ruptures is possible with adequate nonoperative therapy. Similar initial conditions of both groups ensured that our results are related to the intervention. Comparisons are not confounded by differential regression to the mean or bias.

According to the results of IKDC and questionnaire, the majority of STK group patients returned to normal physical activity and a close-to-normal knee function within 3 months of their injury. We could not confirm the results of Costa-Paz et al. that all of the patients with standard nonoperative therapy returned to the same physical activity as before the lesion. In contrast to our followup of 3 months, Costa-Paz observed his patients for 25 months retrospectively [9]. We highly disagree with Noyes et al. who stated that an ACL rupture hinders athletes to continue with their activities [2]. In contrast, as an observation in addition to our clinical results, our patients started sports immediately after one single conservative treatment of their ACL injury with nearly normal range of motion (extension and flexion). Besides spontaneous healing, Khalifa therapy improved their knee function clinically, evaluated by the IKDC score. The KT-1000 test confirms the better knee stability of STK group already on day 1 and after 3 months. Claudication appeared less in the STK group.

Pain was nearly abolished after one treatment in STK group, with an increase in well-being. These effects were long-lasting and improved further during the 3 months of follow-up. Compared to ST, STK patients demonstrated an impressive reduction in days of return to work, making this therapy highly interesting for patients, insurances, and employers.

Last but not least, the MRI confirms an end-to-end continuous ACL with homogeneous signal and disappearance of secondary rupture signs in 47% of group STK. No patient of group ST demonstrated such an ACL restitution. Costa-Paz et al. reported in their retrospective study that all of their 14 patients with ACL rupture were stable after a follow-up of 25 months and had demonstrated spontaneous healing of ACL in MRI [9]. We could not confirm this finding with our shorter follow-up of 3 months. However, we could show that ACL healing is possible within the short period of 3 months with one special treatment, but not with standard conservative physiotherapy.

Manual Khalifa therapy might be a way to accelerate this spontaneous healing. Nevertheless, our results deserve further studies and more frequent clinical application. Although the effect of this study, *d* = 1.6 standard deviations at *t*_1_ or *d* = 2.0 standard deviations after 3 months at *t*_2_ (mean difference of IKDC scores divided by the larger standard deviation, that is, a conservative estimate), was huge. This study had a 98% power to detect such an effect. Hence, it is unlikely that our measured data are the results only by chance.

It is difficult to speculate which patient might have the potential to heal his ruptured ACL spontaneously and who needs ACL reconstruction. We could not confirm Kurosaka's finding that the proximal location of the ACL rupture is associated with better healing potential [12]. In our study, no correlation of the ACLs lesion location and the healing capability could be detected. Perhaps patient's age, activity and comorbidity and maybe some remaining continuous ACL fibres, which cannot be seen in MRI, could determine the clinical potential of spontaneous healing. The remaining eight patients of the group STK still show a complete ACL rupture in MRI at *t*_2_ but demonstrate a functionally stable knee and continue with their athletic activity without any pain or decreased knee function.

Some limitations need to be discussed. (1) Only 30 patients were examined, which might be too few to generalize. However, the impressive clinical difference between both treatment groups confirms its clinical relevance. (2) Sensitivity and specificity of MRI are limited to 85–95% which may lead to wrong positive and wrong negative results [23, 24]. This has to be considered, even if Ihara reported very good validity of MRI to diagnose ACL lesions [3, 11, 25–27]. Arthroscopy as the current diagnostic gold-standard was not authorized by the Ethics Committee because patients reported no pain or limitations after Khalifa therapy. The placebo effect might be a limitation for our results [28]. Maybe placebo analgesia could play a role in the immediate functional improvement [20]. At the moment, it is not clear how this may subsequently aid the recovery process.

Further research needs to clarify the mechanisms and pathways being involved and the generalizability of this method to other therapists trained in manual Khalifa therapy. As a follow-up to this study, an interdisciplinary multi-centre study plans to find explanations for the mechanisms being activated by Khalifa therapy [29]. Physically induced epigenetic effects might be an explanation by modifying stem cell activation [30–37]. In addition, manual Khalifa therapy might directly influence proprioception and biomechanics, which could explain the immediate effects of this therapy [38–40].

We conclude that spontaneous healing of ACL rupture is possible within 3 months after lesion, enhanced by Khalifa therapy. The effect sizes of 1.6 and 2.0 standard deviations after treatment and after 3 months are considerable and prompt further work. Further progress in understanding the underlying mechanisms including placebo will be possible when more experience with the manual pressure therapy has been gathered by other therapists.

## Figures and Tables

**Table 1:** Demographic results (mean values + absolute numbers in brackets) of the study participants with comparable initial conditions (no significant differences).

Variable	ST group	STK group
Men	7	7
Women	8	8
Age	28.8	30.5
BMI	23.4	24.2
Nonsmoker	93% (14)	80% (12)
Economically active (before injury)	87% (13)	80% (12)
Austrian citizenship	100% (15)	93% (14)
Alcohol intake: occasionally	60% (9)	60% (9)
Knee affected (right/left)	7/8	10/5

**Table 2:** Clinical examinations.

Group	Examination dates						
		*t*_0_		*t*_1_		*t*_2_	
		ST	STK	ST	STK	ST	STK
KT-1000-Lachman (side-to-side)	>5 mm	5	6	2	0	0	0
	2–5 mm	9	9	13	6**	12	4*
	<2 mm	1	0	0	9**	2	11**

Deficiency of extension	>10°	1	3	0	0	0	0
	5–10°	8	4	5	0*	0	0
	0–5°	4	7	3	1	5	0*
	0°	2	1	7	14*	10	15*

Maximal flexion	<90°	2	3	1	0	0	0
	90–120°	10	7	6	3*	0	0
	>120°	1	4	6	3*	6	2*
	Free	2	1	2	9**	9	13*

Maximal muscle force	1–5	2.7	2.7	3.1	4.4**	4.1	4.8*

*P < 0.05**P < 0.01 (chi-square-test).

**Table 3:** Pain and well-being (mean and 95% confidence intervals (CI)) of standard group (ST) and standard group + manual Khalifa therapy (STK) at all evaluation dates (*t*_0_, *t*_1_, and *t*_2_).

Group		ST (CI)	STK (CI)
Pain	*t*_0_	3.5 (2.3–4.7)	5.2 (3.9–6.5)
	*t*_1_	3.4 (2.2–4.6)	1.0 (0.4–1.6)
	*t*_2_	2.3 (1.3–3.2)	0.2 (0.03–0.4)

Well-being	*t*_0_	6.3 (5.0–7.5)	6.5 (5.3–7.7)
	*t*_1_	6.2 (5.2–7.2)	7.7 (6.7–8.7)
	*t*_2_	6.9 (6.3–7.4)	9.4 (8.8–9.9)
